# GSK-3*β* at the Intersection of Neuronal Plasticity and Neurodegeneration

**DOI:** 10.1155/2019/4209475

**Published:** 2019-05-02

**Authors:** Tomasz Jaworski, Ewa Banach-Kasper, Katarzyna Gralec

**Affiliations:** Laboratory of Animal Models, Nencki Institute of Experimental Biology PAS, 02-093 Warsaw, Poland

## Abstract

In neurons, Glycogen Synthase Kinase-3*β* (GSK-3*β*) has been shown to regulate various critical processes underlying structural and functional synaptic plasticity. Mouse models with neuron-selective expression or deletion of GSK-3*β* present behavioral and cognitive abnormalities, positioning this protein kinase as a key signaling molecule in normal brain functioning. Furthermore, mouse models with defective GSK-3*β* activity display distinct structural and behavioral abnormalities, which model some aspects of different neurological and neuropsychiatric disorders. Equalizing GSK-3*β* activity in these mouse models by genetic or pharmacological interventions is able to rescue some of these abnormalities. Thus, GSK-3*β* is a relevant therapeutic target for the treatment of many brain disorders. Here, we provide an overview of how GSK-3*β* is regulated in physiological synaptic plasticity and how aberrant GSK-3*β* activity contributes to the development of dysfunctional synaptic plasticity in neuropsychiatric and neurodegenerative disorders.

## 1. Neuronal Plasticity

Neural plasticity is an ability of the brain to adapt in response to normal developmental processes, experience, or injury. It covers such modifications in the brain structures as growth of new neurons, the formation of new networks, and change within existing networks, that is, changes in synaptic strengths, resulting in modifications in function and behavior.

## 2. Synaptic Plasticity

Reversible modification of synaptic strength underlies synaptic plasticity and is activity dependent. Synaptic strength can either be enhanced in a process of long-term potentiation (LTP) or depressed in long-term depression (LTD), and it affects both pre- and postsynaptic sides. LTP is triggered by the intense activation of the NMDA receptor producing a signaling cascade that causes the recruitment of AMPA receptors into the postsynaptic membrane, whereas LTD is triggered by weaker and prolonged activation of NMDA receptors leading to the removal of postsynaptic AMPA receptors [1]. Majority of the excitatory synapses are located on dendritic spines, and their growth following LTP and elimination following LTD are two opposite facts accompanying the bidirectional plasticity of excitatory transmission. Formation of new spines, as well as their morphological modifications in the adult brain, constitutes the structural bases of neuronal plasticity. The dynamic changes of dendritic spine morphology reflect changes in synaptic strength according to its use or disuse. It should be noted, however, that other forms of synaptic plasticity exist which add to the complexity of glutamatergic synapses [2].

On the other hand, inhibitory synaptic transmission driven by the interaction of GABA and ionotropic GABAA receptors constitutes a major form of inhibitory synaptic transmission. Loss of synaptic stability caused by improper excitatory/inhibitory balance and trafficking of synaptic receptors as well as abnormal density and morphology of dendritic spines may lead to the disruption of neuronal circuits resulting in neuropsychiatric disorders. The underlying mechanisms remain to be elucidated, but they depend essentially on kinase-dependent signaling pathways [3, 4].

## 3. Glycogen Synthase Kinase-3

Glycogen Synthase Kinase-3 (GSK-3) is a serine/threonine protein kinase that was first discovered for its role in glycogen synthesis [5]. Later on, extensive studies have implicated GSK-3 in the regulation of many critical cellular processes with over 40 different proteins identified as phosphorylation targets for GSK-3 [6].

GSK-3 exists as two isozymes, GSK-3 alpha (*α*) and GSK-3 beta (*β*), both of which are encoded by distinct genes [7]. They split from the common ancestor at the emergence of vertebrates, while birds lost GSK-3*α* in the evolution [8]. GSK-3*α* and *β* share 85% amino acid sequence similarity, including 98% sequence identity within their catalytic domains [7]. Despite their structural similarity, GSK-3*α* and GSK-3*β* are not functionally identical because the beta isozyme is indispensable in development [9, 10]. In mammals, both GSK-3 isozymes are ubiquitously expressed in all tissues [7], but they are most abundant in the adult brain where they are crucial for its function [11].

GSK-3 is unique among other kinases because it is constitutively active in quiescence cells under resting conditions [12, 13]. The extracellular signals such as growth factors, neurotransmitters and hormones initiate signaling pathways, which cause the reduction of GSK-3 enzymatic activity by dynamic serine phosphorylation of GSK-3. This inhibitory regulation is achieved by a rapid and reversible N-terminal phosphorylation of Ser21 for GSK-3*α* and Ser9 for GSK-3*β*, which creates a pseudosubstrate that binds to the GSK-3 catalytic domain and prevents access of substrates to the GSK-3 active site [12, 14–17].

Phosphorylation and thus inhibition of GSK-3*α*/*β* is carried out by multiple kinases, including Akt/PKB and protein kinases A (PKA) and C (PKC) [6]. In contrast, the dephosphorylation of the N-terminal serine residue by the serine/threonine protein phosphatase 1 (PP1) and protein phosphatase 2A (PP2A) results in the activation of GSK-3 [6, 13, 15, 16].

In contrast, the positive regulation of GSK-3 is achieved by tyrosine phosphorylation: Tyr279 in GSK-3*α* and Tyr216 in GSK-3*β*. Tyrosine phosphorylation in GSK-3 occurs cotranslationally by autophosphorylation or is executed by different tyrosine kinases [18–21].

In the mouse brain, GSK-3*β* exists as three phosphoisotopes: double phosphorylation at Ser9 and Tyr216, single phosphorylation at Tyr216, and the nonphosphorylated isotype, the active form, i.e., phosphorylated at Tyr216 with little Ser9 phosphorylation predominating [22]. In neurons, changes in membrane electrical potential or insulin-like growth factor (IGF) treatment affect GSK-3*β* activity by dynamic PI3K/Akt-mediated phosphorylation and PP2A/PP2B-mediated dephosphorylation of Ser9 [23], while phospho-Tyr216 level remains unchanged [22].

**Figure 1:**
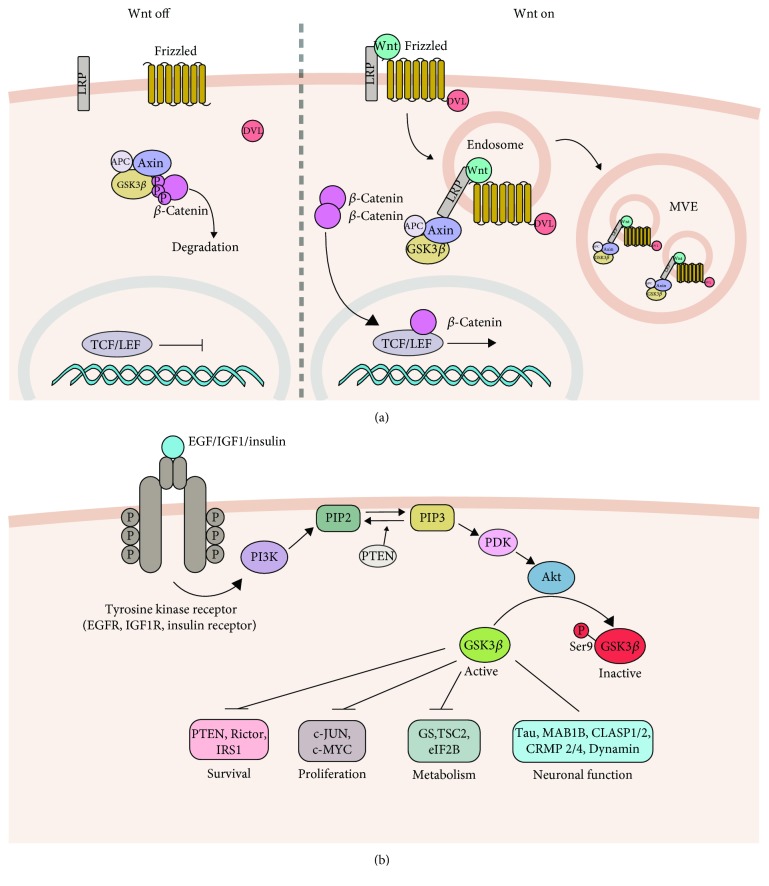
Molecular mechanisms of GSK-3*β* regulation. (a) The Wnt canonical pathway. In the absence of Wnt, *β*-catenin is degraded within a destruction complex composed of Axin, APC, and GSK-3*β* proteins. Following Wnt binding to Frizzled and LRP5/6 receptors, Dvl is recruited resulting in the sequestration of the destruction complex within the MVB. This allows *β*-catenin to accumulate, translocate to the nucleus, and subsequently induce gene expression via the TCF/LEF transcription factors. (b) The PI3K/Akt pathway. The activation of PI3K following the stimulation of Tyrosine Kinase Receptor leads to the production of PIP3. Akt kinase is recruited and is activated upon phosphorylation at Thr308 and Ser473 by PDK1 and mTORC2, respectively. The signal is terminated following PIP3 dephosphorylation by PTEN phosphatase. Akt kinase phosphorylates and inhibits GSK-3*β* activity by a reversible phosphorylation at Ser9. An incomplete list of the GSK-3*β* substrates and cellular processes that it regulates is shown.

Two independently regulated pools of GSK-3 exist in the cell: the Wnt signaling pathway (Figure 1(a)) and the PI3K/Akt signaling pathway (Figure 1(b)). In the Wnt signaling pathway, in the absence of extracellular Wnt ligands or the presence of Wnt negative modulators such as extracellular protein Dickkopf-1 (DKK1), the transcriptional coactivator *β*-catenin is phosphorylated by GSK-3 in a complex composed of the tumor suppressor adenomatous polyposis coli (APC) and the scaffolding protein Axin. Subsequently, phosphorylated *β*-catenin is targeted for proteasome-dependent degradation. In the presence of extracellularly secreted Wnt proteins, Frizzled receptor and the low-density lipoprotein-related protein 5 and 6 (LRP5/6) receptors are activated [24]. This event leads to the recruitment of Dishevelled mammalian homolog Dvl1, resulting in the destabilization of the Axin-APC-GSK-3*β* protein complex and its sequestration into multivesicular bodies (MVB) [25]. GSK-3 inactivation allows for *β*-catenin stabilization and facilitates gene expression by the TCF/LEF transcription factors.

In the phosphoinositide 3-kinase (PI3K)/Akt pathway, growth signals activate the catalytic subunit of PI3K, which phosphorylates phosphatidylinositol-4,5-bisphosphate (PIP2) to produce phosphatidylinositol-3,4,5-trisphosphate (PIP3) and activates phosphoinositide-dependent protein kinase-1 (PDK-1). PDK-1 phosphorylates and thus activates the recruited serine-threonine kinase Akt/protein kinase B. Akt/PKB phosphorylates GSK-3 to inhibit its activity [6, 12, 15].

GSK-3 controls many neuronal functions by phosphorylating protein substrates involved in the regulation of gene transcription, metabolism, apoptosis, and cytoskeletal dynamics (Figure 1(b)). To ensure the proper execution of these actions, GSK-3 activity must be accurately controlled by the interplay of phosphorylation, localization, and sequestration by GSK-3-interacting proteins [6, 26, 27].

## 4. GSK-3 Function in the Developing and Adult Brain

### 4.1. Neuronal Progenitors: Proliferation and Differentiation

Neural progenitor proliferation and differentiation are regulated by multiple extracellular signals and intracellular signaling mechanisms in which GSK-3 is implicated. Early in neural development, GSK-3 functions to regulate neural progenitor self-renewal, homeostasis, and apical-basal polarity via *β*-catenin, Notch, FGF, and Wnt signaling [28].

Establishing neuronal polarity is a consequence of the reorganization of cytoskeletal elements after the local activation of symmetry-breaking signals. GSK-3 is a key regulator of neuronal polarity and microtubule-cytoskeleton reorganization [29, 30]. These functions are controlled by GSK-3-mediated phosphorylation of microtubule-associated proteins (MAPs), such as collapsin response mediator protein-2 (CRMP-2) [31], adenomatous polyposis coli (APC) [32], Tau [33], microtubule-associated protein 1B (MAP1B) [34], Doublecortin (DCX) [35], end-binding 1 (EB1) [36], and cytoplasmic linker-associated proteins (CLASPs) [37], and subsequent regulation of cytoskeletal dynamics. For example, APC and CLASPs promote microtubule stability and, upon phosphorylation by GSK-3, they dissociate from and destabilize microtubules [37, 38]. Therefore, polarized deposition of polarity proteins underlies asymmetric cell division which is necessary for the neurogenic division of neural progenitors. Indeed, polarized apical deposition of polarity proteins, including APC, EB1, and cadherin, is disrupted in GSK-3*α*/*β*-deleted developing cortex [28].

### 4.2. Neuronal Migration

Following differentiation of progenitors into neurons, GSK-3 signaling is crucial to neuronal migration. For example, removal of GSK-3*α* and GSK-3*β* in cortical excitatory neurons leads to the failure of radial migration in the cortex [39]. GSK-3 regulates neuronal migration by phosphorylating key microtubule regulatory proteins such as APC and other microtubule-associated proteins to rearrange the intracellular cytoskeleton. As mentioned before, APC is a microtubule-associated protein and is important for microtubule-based cytoskeleton dynamics [40]. When GSK-3 is inactive, APC stabilizes microtubules at the leading edge of migrating neurons [38]. When GSK-3 becomes active, it binds to and phosphorylates APC causing its dissociation from microtubules [41].

Other studies have implicated other GSK-3 interacting proteins, including *β*-catenin and DISC1, in neuronal migration [42–46]. DISC1/GSK-3 interaction may be particularly important for determining the transition of neural progenitor self-renewal to neuronal migration because GSK-3 binds to DISC1 during the embryonic stage (E14) when neural progenitor proliferates but dissociates from DISC1 during later embryonic stages (E18) when neuronal migration takes place [46].

### 4.3. Neuronal Morphology and Synaptic Development

Several lines of evidence implicate GSK-3 in the regulation of different aspects of neuronal morphogenesis, including axon growth, dendritic branching, and synaptic development. Pharmacological inhibition of GSK-3 decreases the rate of axon elongation, increases the size of growth cones [47], and disturbs polarity, leading to the formation of multiple axon-like processes in hippocampal neurons [48, 49]. Likewise, genetic elevation of GSK-3*β* activity causes shrinkage of dendrites, whereas GSK-3*β* inhibition enhances dendritic growth *in vivo* [50]. Another study showed that neurons with deleted GSK-3 exhibit markedly abnormally oriented basal dendrites [39].

**Figure 2:**
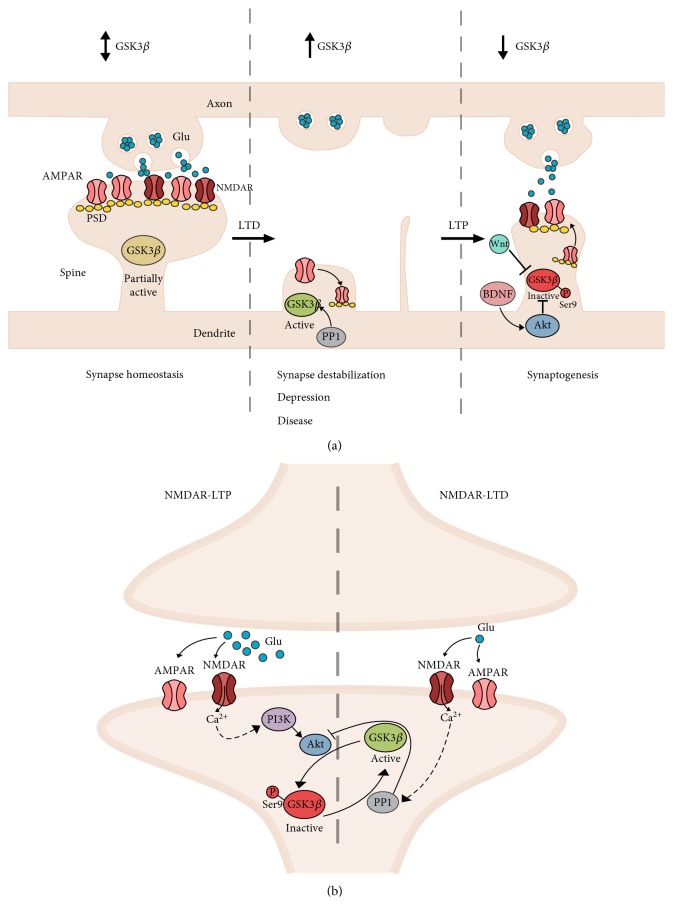
GSK-3*β* at glutamatergic synapse. (a) Role of GSK-3*β* in the structural plasticity of glutamatergic synapse. (Left) Under normal conditions, synapse function is maintained by homeostatic mechanisms that depend on the cycling of glutamate receptors within the synapse. Transient changes in GSK-3*β* activity will support molecular mechanisms required for these processes. (Middle) Synaptic destabilization following LTD or chronic stress decreases synaptic density and causes synapse atrophy. High GSK-3*β* activity is required for pre- and postsynaptic molecular mechanisms to support the occurrence of LTD. Increased GSK-3*β* activity has been reported in different neurological and neuropsychiatric disorders. (Right) Following LTP stimuli, GSK-3*β* is inhibited to enable synaptic growth. LTP stimuli also increase BDNF and Wnt proteins which act to inhibit GSK-3*β* during LTP. (b) GSK-3*β* determines the direction of NMDA receptor-mediated plasticity. (Right) During LTD, activation of PP1 causes dephosphorylation and thus activation of GSK-3*β* by the Ser9 mechanism. Simultaneously, active PP1 inhibits Akt preventing Ser9 phosphorylation of GSK-3*β*. During LTP, the activation of NMDA receptors stimulates the PI3K-Akt pathway, which phosphorylates and inhibits GSK-3*β* activity to prevent the induction of LTD.

GSK-3 also contributes to the regulation of synapse morphology and formation in mature, postmitotic neurons (Figure 2(a)). Deletion of the GSK-3*β* gene in the cortex and hippocampus causes a reduction of spine density, loss of persistent spines, and reduced stabilization of new spines, accompanied by a decrease of AMPAR-dependent miniature excitatory postsynaptic currents [51]. Accordingly, overexpression of GSK-3*β* alters dendritic branching and reduces the number of the functional synapses of dentate gyrus granule neurons [52]. A recent study showed that GSK-3*β* is involved in the maturation of dendritic spines, because genetically elevating GSK-3*β* activity increases the number of thin spines, whereas removal of the GSK-3*β* gene increases the number of stubby spines in the dentate gyrus neurons [53]. Likewise, pharmacological inhibition of GSK-3*β* decreases the number of mature spines favouring an accumulation of immature types [54].

### 4.4. Neurotransmission

GSK-3*α* and *β* are present within the synapse because they were detected in the synaptosomal fraction which consists of pre- and postsynaptic termini [55]. More specifically, an electron microscopic study showed GSK-3*β* labelling of postsynaptic densities in a subset of dendritic spines [56].

GSK-3 plays an important role in synaptic plasticity at GABAergic as well as at glutamatergic synapses. At GABAergic synapses, active GSK-3*β* decreases inhibitory synaptic strength [50] by phosphorylating the scaffolding protein gephyrin [57].

At glutamatergic synapses, GSK-3*β* regulates the interaction between two major forms of synaptic plasticity: NMDA-dependent LTP and LTD (Figure 2(b)). During LTP, the activation of NMDA receptors causes the inhibition (by Ser9 phosphorylation) of GSK-3*β* activity via the PI3K/Akt pathway, whereas the action of PP1 in LTD causes an increase of GSK-3*β* activity [58]. Thus, GSK-3*β* is crucial for the initiation of NMDA-induced LTD in hippocampal neurons.

Molecular mechanisms requiring the modulation of GSK-3 Ser21/9 phosphorylation, during experimental LTP or LTD, are crucial for learning and memory [55, 58, 59]. The phosphorylation of GSK-3*β* at Ser9 increases following the training of mice in hippocampus-dependent cognitive tasks, i.e., inhibitory avoidance and novel object recognition task [59]. Furthermore, LTP is impaired, whereas LTD is facilitated, in two different transgenic mice overexpressing active GSK-3*β* [55, 59]. These LTP deficits can be reversed by treatment with lithium, a GSK-3 inhibitor [55]. Accordingly, removal of GSK-3*β* in dentate gyrus excitatory neurons inhibits hippocampal synaptic transmission and reduces levels of NMDAR and AMPAR receptors, postsynaptic PSD93 and drebrin, and presynaptic synaptophysin proteins causing impairments in spatial and fear memories [60].

Furthermore, GSK-3 contributes to NMDA and AMPA receptor trafficking and function in cortical neurons [61, 62]. GSK-3 causes internalization of NMDARs and forms a complex between AMPARs, thereby affecting the expression of LTD. AMPA receptor mobilization is important for LTD to occur. A critical step in this process is the destabilization of PSD-95 by GSK-3*β* [63].

In addition to postsynaptic actions, GSK-3 also participates in presynaptic functions in developing and mature synapses [64]. For example, high GSK-3 activity reduces glutamate release from the presynapse causing impairments in LTP [55, 65]. Additionally, retrieval of synaptic vesicles at the presynapse by endocytosis requires the regulation of dynamin 1 by GSK-3 [66]. Moreover, GSK-3*β* negatively regulates synaptic vesicle fusion events via interfering with Ca(2+)-dependent SNARE complex formation which is required for efficient neurotransmitter release [67]. These observations show that GSK-3 is crucial for synapse assembly and function, although the GSK-3 synaptic phosphoproteome has not been described yet. Overall, GSK-3 regulates neuronal excitation/inhibition balance. Dysregulated excitatory/inhibitory control has been reported in different neuropsychiatric disorders.

## 5. Implications of GSK-3 Dysregulation

### 5.1. GSK-3 Knockout and Transgenic Mouse Models

The dysfunction of GSK-3 signaling pathways is associated with the pathogenesis of different neurological and neuropsychiatric disorders. Several mouse models lacking or overexpressing GSK-3*α* or *β* have been generated that mimic pathological conditions observed in different neuropsychiatric and neurological disorders. These mice recapitulate pathological conditions with aberrant GSK-3 activity and thereby point at GSK-3 as a critical regulator of different physiological neurological processes.

Total removal of GSK-3*β* is lethal in late embryonic development due to liver apoptosis or heart defects [9, 10]. Removal of only one GSK-3*β* allele causes behavioral abnormalities, including aggressive behaviors, increased anxiety, and memory deficits, in GSK-3*β* heterozygous (+/-) mice [68, 69].

In contrast, homozygous mice lacking GSK-3*α* are viable but male mice are infertile [70]. They show minor abnormalities in brain anatomy, such as an altered neuronal architecture of the hippocampus [70] or a lower number of Purkinje cells in the cerebellum [71]. These two mouse strains show minor neurobehavioral abnormalities such as reduced exploratory activity, increased anxiety, and decreased social motivation and associative memory [70, 71].

Postnatal neuronal specific GSK-3*β* knockout mice (GSK-3*β*^n-/-^) together with GSK-3*α* mice (GSK-3*α*^n-/-^) were developed based on the Cre/loxP system to omit the developmental problems of GSK-3*β* deficiency [72]. Neurological examination showed that GSK-3*β*^n-/-^ mice have reduced dentate gyrus volume [73] and decreased stability of dendritic spines [53], while GSK-3*α*^n-/-^ mice have a reduced-size CA1 pyramidal blade and pre- and postsynaptic deficits [70], suggesting distinct synaptic functions of GSK-3 isozymes in the adult brain.

Transgenic mice that overexpress human GSK-3*β* employ the S9A mutant form of the kinase to prevent its inhibitory phosphorylation [74]. The thy1 gene promoter employed drives the expression of GSK-3*β*(S9A) postnatally in neurons. This transgenic mouse displays a twofold higher GSK-3*β* level and activity relative to wild-type mice. Consequently, increased tau phosphorylation is evident, but only in older GSK-3*β*(S9A) mice. These mice have decreased brain weight and volume counterbalanced by a higher cortical neuronal density and decreased size of their cell bodies and of their somatodendritic compartments [75]. The decreased brain size was further confirmed in a recent study showing a decreased dentate gyrus volume in GSK-3*β*(S9A) mice [73]. Biochemical analysis showed increased brain-derived neurotrophic factor (BDNF) and Akt1 levels in the hippocampus and decreased levels of PPP2R3A (PP2A regulatory subunit) and GSK-3*α* in the striatum in GSK-3*β*(S9A) mice [76]. Furthermore, overexpression of GSK-3*β* was shown to result in the differential expression of a large number of proteins, including the downregulation of MAP2 [77]. Despite increased tau phosphorylation and decreased hippocampus volume, GSK-3*β*(S9A) mice display normal memory in the Morris water maze test [74]. However, follow-up studies demonstrated impairments in hippocampal-dependent, species-typical behavioral tasks [73] and passive inhibitory avoidance [59]. Furthermore, GSK-3*β*(S9A) mice show hyperactivity and lower immobility time in the forced swim test (FST) which recapitulate symptoms of schizophrenia or the manic phase of bipolar disorder [76].

These mouse studies show that while GSK-3*β* is important during development, in the adult brain both GSK-3 isozymes have important nonredundant functions in the regulation of learning, memory, and behavior, which may result from similar but not the same spectrum of protein substrates in neurons [78].

Altogether, a delicate balance of GSK-3*β* activity is important for the regulation of different aspects of neuronal plasticity at the developmental stage as well as in adulthood. Not surprisingly, the dysregulation of GSK-3*β* activity may have deleterious consequences leading to brain disorders.

### 5.2. Alzheimer's Disease

Alzheimer's disease (AD) is characterized by a progressive loss of episodic memory and by cognitive and behavioral impairments and ultimately death. Synaptic dysfunction and hence memory impairments come early in the disease process. Histopathological hallmarks at postmortem analysis are extracellular senile plaques made up of amyloid-*β* (A*β*) protein and intracellular neurofibrillary tangles (NFTs) composed of hyperphosphorylated tau protein. Since its initial discovery as a tau protein kinase [79], GSK-3*β* is considered to be essential to AD pathogenesis [80]. It plays a fundamental role in pathological events such as Tau phosphorylation, A*β* formation, neurotoxicity, neuritic dystrophy, impaired cognition, neuronal survival, and neurodegeneration [74, 81–84]. Increased levels of GSK-3 have been reported in brains from AD patients compared to age-matched control samples [85]. Furthermore, a spatial and temporal pattern of increased active GSK-3*β* expression correlates with the progression of neurofibrillary tangles (NFT) composed of hyperphosphorylated forms of Tau, A*β* formation, inflammatory markers, and neurodegeneration [86]. Accordingly, increased GSK-3*β* activity has been used to replicate neuronal dysfunctions in mouse models of AD [74, 84]. GSK-3*β* has been shown to be the major tau kinase *in vivo* [74], and it phosphorylates at least 36 residues in tau protein [87]. Furthermore, comparative phenotypic analysis of two bigenic mouse lines APP.V717I-tau.P301L and GSK-3*β*.S9A-tau.P301L reveals that amyloid or GSK-3*β* leads to a similar tau phosphorylation pattern and NFT accumulation [84]. Additionally, A*β* has been shown to activate GSK-3*β* signaling *in vitro* [88]. Altogether, GSK-3*β* is the mediator of amyloid action on tau phosphorylation and neurodegeneration in AD.

It should be noted that changes in the GSK-3*β* kinase activity, besides being involved in the regulation A*β* or tau phosphorylation, will negatively affect synaptic plasticity essential for learning and memory [55, 58, 59, 65]. For example, overexpression of GSK-3*β* in transgenic mice impairs memory [89, 90]. Pharmacologically balancing normal levels of GSK-3*β* activity recues memory deficits [90, 91]. This GSK-3*β*-induced cognitive impairment is mediated by tau protein because the genetic deletion of tau as well as GSK-3*β* inhibition blocks A*β*-induced impairments of LTP [92]. Furthermore, the genetic deletion of tau in GSK-3*β*-overexpressing mice ameliorates memory deficits [93].

### 5.3. Parkinson's Disease

Parkinson's disease (PD), the second most common neurodegenerative disease, is a chronic movement disorder resulting from the progressive loss of dopaminergic neurons in the substantia nigra pars compacta, leading to pathological and clinical abnormalities, including bradykinesia (slowness and minimal movement), rigidity, resting tremor, and postural instability. Additional symptoms include cognitive decline, depression, anxiety, and sleep disturbances resulting from neurodegeneration in the cortex and brainstem [94, 95]. The loss of dopaminergic neurons and thus decreased dopamine levels in the striatum is accompanied by an intracellular buildup of alfa-synuclein inclusions called Lewy bodies (LB) and hyperphosphorylated tau [96].

Evidence for GSK-3*β* involvement in PD comes from genetic studies in which single-nucleotide polymorphisms (SNPs) in the *GSK-3β* gene (rs334558 and rs6438552) are associated with PD [97]. The T allele (rs6438552) alters the GSK-3*β* splicing pattern resulting in the augmentation of GSK-3*β* activity [97]. Other studies in different populations have also linked SNPs in the *GSK-3β* gene to PD [98–100].

Accordingly, an increased GSK-3*β* expression has been reported in postmortem PD brains [101]. Furthermore, GSK-3*β* colocalizes with *α*-Synuclein in the Lewy bodies (LBs) [101]. *In vitro* GSK-3*β* phosphorylates *α*-Synuclein at Ser129 facilitating its toxic misfolding, aggregation, and accumulation leading to the degeneration of dopaminergic neurons [102]. Furthermore, GSK-3*β* contributes to Tau pathology associated with PD [102], corroborating the genetic data [100, 103]. Specifically, in a cell model of PD alpha-synuclein, pSer396/404-Tau and pGSK-3*β* coimmunoprecipitate following MPP(+) treatment [104]. Moreover, GSK-3*β* inhibitors prevent MPP(+)-induced death, increased *α*-synuclein accumulation, and pTau formation [104]. Studies from animal models demonstrated that in mice expressing a constitutively active, human GSK-3*β*(S9A) mutated form, levels of p-*α*-synuclein-S129 and pTau (S396/404) rise in TH+ dopaminergic neurons along with animal aging [102]. In *α*-synuclein A53T mutant mice, elevated levels of *α*-synuclein together with increased levels of pTau (pSer202, 396/404) and the active form of pGSK-3*β* (pTyr216) were detected in the striatum by western blot analysis; all of these components were also found to aggregate together, as confirmed by immunohistochemical stainings [105].

In line with these results, GSK-3*β* inhibitors were considered to counteract the degeneration of dopaminergic neurons. Accordingly, chronic treatment with lithium prevented the degeneration of dopaminergic neurons in the mouse model of PD [106]. Likewise, more specific GSK-3*β* inhibitors such as indirubin-3′-oxime and AR-A014418 suppress the loss of dopaminergic neurons and restore dopamine concentration [107].

Cautiously, human study demonstrated that chronic lithium treatment itself can induce parkinsonian pathological features, including impaired motor coordination accompanied by neuronal loss in the basal ganglia [108]. Therefore, considerations such as designing specific GSK-3 inhibitors, preventing their side effects, and determining optimum levels of GSK-3*β* inhibition have to be taken into account in planning GSK-3-based therapeutic strategies.

### 5.4. Lithium: GSK-3 Inhibitor

For many years, lithium has been used as a mood stabilizer in the treatment of mental disorders, including bipolar disorder, schizophrenia, and depression. Despite that many molecular targets have been identified, lithium is best known as a GSK-3 inhibitor [109, 110]. Lithium directly inhibits GSK-3*α* and GSK-3*β* [109] both in cells [110] and in the brain *in vivo* [111] at an IC50 of 2 mM, which is slightly higher than the therapeutic concentration of 0.5-1.5 mM [109]. The direct mechanism by which Li^+^ ions inhibit GSK-3 is that they compete for the binding of magnesium, which is a cofactor of different kinases, including GSK-3 [112]. Lithium can also indirectly inhibit GSK-3 by activating the Akt kinase or by disrupting the *β*-arrestin complex [113–115].

A large number of studies on the effects of lithium confirmed that GSK-3 is associated with different diseases, including fragile X syndrome (FXS) and schizophrenia. Lithium or the specific pharmacological modulation of GSK-3 activity has been shown to correct behavioral deficits in mouse models of these diseases [116, 117]. This highlights GSK-3 as a valid target of lithium; however, it must be noted that lithium is a nonspecific GSK-3 inhibitor (it inhibits many other kinases) with high *in vivo* toxicity.

### 5.5. Fragile X Syndrome

Patients with FXS have intellectual disability. Fragile X syndrome (FXS) results from the lack of expression of the functional fragile X mental retardation protein (FMRP) due to the expansion of CGG triplets resulting in the overmethylation of the gene promoter. FMRP is an RNA-binding protein that controls cellular mRNA translocation.

Since mRNA translocation towards dendrites and local translation play a pivotal role in neuronal function, FXS is characterized by several behavioral and brain structural abnormalities. Mice lacking the FMRP expression (FMRP KO mice), which model FXS, display similar characteristics as patients with FXS. FMRP KO mice exhibit impaired structural synaptic plasticity characterized by an increased dendritic spine length and number, accompanied by a reduced maturation of spines, as compared to control mice [118–120]. Indeed, other reports showed that FMRP plays a role in the normal maturation of synaptic connections [118, 121]. In addition, FX mice display distinct functional synaptic alternations such as enhanced metabotropic glutamate receptor- (mGluR-) dependent long-term depression (LTD) in the hippocampal CA1 neurons. Interestingly, further research showed aberrant mGluR signaling to GSK-3 in FX mice, and lithium treatment normalized increased mGluR-dependent LTD at CA1 synapses in these mice [122].

GSK-3 inhibition following the administration of lithium or more specific inhibitors in these mice led to corrections of multiple functional and structural FX-related phenotypes, such as normalization of hyperactive locomotor and social behaviors and improvement of passive avoidance learning as well as normalization of dendritic spine length and density and synaptic transmission [116, 123].

### 5.6. Schizophrenia

Schizophrenia is a widespread mental disorder, characterized by progressive functional decline and lifelong disability. Common symptoms are typically categorized into positive (hallucinations and delusions), negative (disruption of normal emotions and behavior), and cognitive (disruption of executive performance and memory). People with schizophrenia often have additional mental health problems such as anxiety or depression. Schizophrenia is thought to be caused by a combination of environmental and genetic factors.

Genetic studies have supported the association between *AKT1* genetic variants and schizophrenia [124, 125], suggesting that impaired AKT/GSK-3 signaling contributes to the pathogenesis of schizophrenia [125, 126]. AKT1 protein level is significantly reduced in the hippocampus and frontal cortex in postmortem brain samples. Consequently, the activity of the major AKT1 target—GSK-3—is altered in patients with schizophrenia [125]. Additionally, *GSK-3β* promoter polymorphism rs3755557 that results in a higher promoter activity [127] is associated with schizophrenia in the Chinese population [128].

A recent study showed that increased GSK-3*β* activity early in development predisposes to altered synaptic plasticity, dendritic spine loss, and cognitive disability in a rat neurodevelopmental model of schizophrenia [54]. Accordingly, chronic treatment with antipsychotics such as clozapine, risperidone, or haloperidol increases the inhibitory phosphorylation of GSK-3*β* in the rat prefrontal cortex and striatum [129, 130].

Dysregulated dopamine neurotransmission is thought to underlie schizophrenia pathophysiology as dopamine D2 receptor antagonists are antipsychotic drugs. Akt/GSK-3 signaling is important for dopamine D2 receptor function, because mice lacking GSK-3*β* have an impaired function of the striatal D2 receptor [131]. Molecularly, the D2 receptor stimulates the formation of a signaling complex made up of *β*-arrestin-2, Akt, and PP2A—the latter inactivates Akt by the dephosphorylation of its regulatory Thr308 residue [132]. Accordingly, the regulation of Akt by dopamine is impaired in mice devoid of *β*-arrestin-2 [132]. Akt inhibition is known to activate GSK-3, suggesting that GSK-3 signaling is involved in the regulation of dopamine-dependent locomotor behavior. Likewise, pharmacological or genetic abolishing of GSK-3 activity decreases dopamine-dependent locomotor behavior [133].

### 5.7. Major Depressive Disorder

Major depressive disorder (MDD) is the most frequent psychiatric disorder with a prevalence of 17% in the general population, although gender disproportion exists [134]. MDD negatively affects personal life and general health. The most widely used animal model of depression is the chronic unpredictable mild stress (CUMS) model in rats. CUMS results in the augmentation of GSK-3*β* activity [135–137]. Accordingly, lithium and specific GSK-3*β* inhibitors ameliorate cognitive deficits induced by CMS [135–137].

One of the associated symptoms of MDD are disturbances in the hypothalamic–pituitary–adrenal axis (HPA axis) connected with an incorrect response of the glucocorticoid receptor to chronic stress [138]. Chronic administration of corticosterone that models depression in mice impairs synaptic plasticity and upregulates GSK-3*β* activity—both of which are ameliorated by the administration of an antidepressant drug [139].

The *GSK-3β* gene may have a role in determining regional grey matter (GM) volume differences in MDD. Analysis of single-nucleotide polymorphisms (SNPs) of *GSK-3β* with regional GM volume differences in patients with MDD showed the most significant association for rs6438552 [140]. In a different study, the activating allele T of the functional polymorphism rs334558 was significantly associated with remission in MDD [141].

### 5.8. Bipolar Disorder

Bipolar affective disorder is characterized by manic episodes that are interspersed with depression. Inadequate serotonin (5HT) neurotransmission may be a key factor driving depression. Evidence suggests that increased serotonergic activity following the administration of antidepressants inhibits GSK-3*β* in the brain by the Ser9 mechanism [142]. Thus, GSK-3*β* may not be properly inhibited in conditions of decreased 5HT levels in depression. Indeed, lower phosphorylated GSK-3*β* Ser9 levels were detected in platelets of patients with schizophrenia [143]. Indeed, animal studies provide further support that overactive GSK-3 contributes to depression. Transgenic mice with GSK-3*β* overexpression show increased locomotor activity as seen in the manic phase of bipolar disorder [76]. Furthermore, the administration of the GSK-3*β* peptide inhibitor, ATP competitive inhibitor, and lithium and the genetic reduction of GSK-3*β* in GSK-3*β*^+/-^ mice produce antidepressant behavioral effects, such as decreased immobilization time in FST, which is indicative of depressive behavior [69, 144, 145].

### 5.9. Epilepsy

Epilepsy, which is estimated to affect over 50 million people worldwide, comprises a group of neurological diseases characterized by epileptic seizures resulting from an excessive neuronal activity [146]. In addition to seizures, epilepsy is usually associated with cognitive impairments. Epilepsy frequently accompanies various mental conditions, such as autism spectrum disorders or schizophrenia. Development of epilepsy, known as epileptogenesis, may take months or even years following brain injury, stroke, brain tumors, brain infections, or birth defects, whereas a small proportion of the cases are due to genetic mutations [147, 148]. Epileptogenesis can be reproduced in animal models using electrical or chemical kindling with pentylenetetrazole (PTZ), whereas the status epilepticus is induced by kainic acid (KA) or pilocarpine [149]. Even though extensive research shows that GSK-3 contributes to brain excitability and seizure-induced pathology, the existing data are conflicting [150–153]. For example, GSK-3*β* phosphorylation at Ser9 was reported to increase or decrease in brain tissue extracted from epileptic patients [154, 155]. Furthermore, kainic acid- (KA-) triggered epileptogenesis was shown to either increase or inhibit GSK-3*β* activity [152, 156]. Acute PTZ injection rapidly increases GSK-3*β* Ser9 phosphorylation and PTZ-induced kindling also gradually increases phosphorylation at Ser9 [53, 151], whereas pilocarpine-induced seizures transiently inactivate GSK-3*β* [150]. Pharmacological studies aimed at elucidating the role of GSK-3*β* inhibition in epilepsy showed a neuroprotective effect of GSK-3*β* inhibition against glutamate-induced toxicity *in vitro* and *in vivo* [157]. Accordingly, the GSK-3*β* inhibitor TDZD-8 protects against seizure-induced damage [152]. Consistently, a recent study reported the anticonvulsant properties of two distinct GSK-3 inhibitors (Indirubin and BIO-acetoxime) in three different animal models of epilepsy: the PTZ-treated zebrafish, the pilocarpine rat model for limbic seizures, and the 6 Hz refractory seizure mouse model [158]. In contrast, lithium was shown to exert proconvulsive [159] or anticonvulsive effects [160].

More complexity comes from recent animal studies. It was shown that genetically increasing as well as decreasing the activity of GSK-3*β* exacerbated seizure-induced brain damage after KA injection into the amygdala [161]. In a different study, GSK-3*β* decreased the susceptibility to kainic acid-induced epileptiform discharges and the progression of kainic acid-induced epileptogenesis [162]. Similarly, the neuronal deficiency of GSK-3*β* exacerbated the magnitude and severity of PTZ-induced seizures in GSK-3*β*^n-/-^ mice (with postnatal neuronal deficiency) [53].

Regardless of these discrepancies, GSK-3*β* is considered an important contributor to the development of epilepsy.

## 6. Conclusions

Evidence convincingly shows that GSK-3*β* is critically involved in various aspects of brain function starting from early brain development, to distinct aspects of its function in the adult such as proper synaptic development and neurotransmission. GSK-3*β* is regulated at multiple levels and precise balance of its activity is important to execute its functions in neurons. Not surprisingly dysregulation of GSK-3*β* activity either in the early development or in the adulthood may predispose to neuropsychiatric and neurological disorders. GSK-3*β* is thus a relevant target for treatment of these diseases. Few GSK-3 inhibitors are currently undergoing clinical trials for various disorders such as progressive supranuclear palsy, Alzheimer's disease or cancer [163]. Pharmacological targeting of this kinase, however, may be problematic because of its involvement in different signaling pathways as well as because of overlapping functions with GSK-3*α* isozyme. Therefore, generating novel inhibitors with increased specificity, designing co-treatments and preventing side effects are of importance in pharmacological targeting of GSK-3.
